# Violence Against Health Workers Amidst Brain Drain in Nigeria: An Issue of Concern

**DOI:** 10.34172/ijhpm.2024.7988

**Published:** 2024-01-31

**Authors:** Moriam Adesola Adegbite, Yusuf Babatunde

**Affiliations:** Faculty of Pharmaceutical Sciences, University of Ilorin, Ilorin, Nigeria.

## Dear Editor,

 The Nigerian Medical Association made a press release on January 2, 2023 on the murder of a physician at his workplace.^1^ Workplace violence and harassment are work-related physical, verbal, sexual, and psychological abuse, threat, harassment and assault of persons at work or on duty.^2,3^ In many countries in the world, scenarios of health workers violence has been documented. The World Health Organization (WHO) records that 52% of health workers have experienced a form of workplace violence, with verbal abuse being the most prevalent of non-physical violence.^3^

 In a study conducted by Usman et al in Kaduna metropolis of Northern Nigeria, 64.4% of health workers who participated in the study has experienced workplace violence.^4^ Similarly, 66.1% of health workers reported to have witnessed at least one case of a colleague’s harassment in the hospital in Southwest Nigeria.^5^ In Southeast Nigeria, 49.7% of the respondents experienced psychological workplace violence in the past year.^6^

 Violence against these health professionals are perpetrated mostly by patients or their relatives and ranges from verbal abuse to physical violence including murder such as that reported by the Nigeria Medical Association in January 2023.^1,4,5^ Incidence of sexual abuse however has the least occurrence.^4^ Although reasons for violence are not justifiable, they are perceived to be caused by long waiting times, lack of bed space and drugs, attitude of the health worker or absence from the duty post during night shift or call hours.^4^

 With the continuous exodus of health professionals from Nigeria, experiencing workplace violence in addition constitutes a double crisis for the already depleted Nigerian healthcare system. Health delivery is hampered by brain drain of the public healthcare workforce and this brain drain is attributed to job dissatisfaction caused by inadequate remuneration, lack of career growth opportunities, and inadequate health infrastructures, etc.^7^ The consequences of workplace violence are not only limited to work injuries leading to absenteeism and job dissatisfaction, but can also aggravate the current brain drain concern.^2^ Thereby impacting the healthcare delivery negatively.

 It is therefore necessary that health organizations put into place measures to ensure that its personnel are protected. Staffs should be trained on how to identify risks of violence, recognize violent incidents, conflict resolution and maintain physical and emotional balance. There should be appropriate measures to improve the work culture such as providing optimal staffing levels, reducing patient waiting time, providing timely information to patients and their relatives, restricting public movement in health facilities and alerting security personnel when violence is threatened.^3^ Hospitals and healthcare institutions need to also make efforts in improving the relationship between health personnel and patients and relations through effective communication channels. Lastly, there should be adequate laws put in place as penalties for any form of workplace violence against health workers spanning from heavy fines to prison sentences in Nigeria.

## Ethical issues

 Not applicable.

## Competing interests

 Authors declare that they have no competing interests.

## References

[1] Sahara Reporters. Nigerian Medical Association Condemns Murder of Doctor in Delta by Relations of Patient on New Year’s Eve. January 2, 2023. https://saharareporters.com/2023/01/02/nigerian-medical-association-condemns-murder-doctor-delta-relations-patient-new-years.

[2] Mento C, Silvestri MC, Bruno A (2020). Workplace violence against healthcare professionals: a systematic review. Aggress Violent Behav.

[3] World Health Organization. Violence and Harassment. https://www.who.int/tools/occupational-hazards-in-health-sector/violence-harassment.

[4] Usman NO, Dominic BO, Nwankwo B, Nmadu AG, Omole NV, Usman OA (2022). Violence towards health workers in the workplace: exploratory findings in secondary healthcare facilities in Kaduna metropolis, Northern Nigeria. Babcock Univ Med J.

[5] Abodunrin OL, Adeoye OA, Adeomi AA, Akande TM (2014). Prevalence and forms of violence against health care professionals in a South-Western city, Nigeria. Sky J Med Med Sci.

[6] Chinawa AT, Ndu AC, Arinze-Onyia SU (2020). Prevalence of psychological workplace violence among employees of a public tertiary health facility in Enugu, Southeast Nigeria. Niger J Clin Pract.

[7] Akinwale OE, George OJ (2023). Personnel brain-drain syndrome and quality healthcare delivery among public healthcare workforce in Nigeria. Arab Gulf J Sci Res.

